# Coarse-Grained Simulation of Mechanical Properties of Single Microtubules With Micrometer Length

**DOI:** 10.3389/fmolb.2020.632122

**Published:** 2021-02-15

**Authors:** Jinyin Zha, Yuwei Zhang, Kelin Xia, Frauke Gräter, Fei Xia

**Affiliations:** ^1^ School of Chemistry and Molecular Engineering, East China Normal University, Shanghai, China; ^2^ Division of Mathematical Sciences, School of Physical and Mathematical Sciences, Nanyang Technological University, Singapore, Singapore; ^3^ School of Biological Sciences, Nanyang Technological University, Singapore, Singapore; ^4^ Interdisciplinary Centre for Scientific Computing (IWR), Heidelberg University, Heidelberg, Germany; ^5^ Heidelberg Institute for Theoretical Studies (HITS), Schloβ-Wolfsbrunnenweg 35, Heidelberg, Germany; ^6^ Max Planck School Matter to Life, Jahnstraβe 29, Heidelberg, Germany; ^7^ Shanghai Engineering Research Center of Molecular Therapeutics and New Drug Development, NYU-ECNU Center for Computational Chemistry at NYU Shanghai, Shanghai, China

**Keywords:** microtubule, persistence length, ultra-coarse-grained model, mechanical property, convolutional and K-means coarse-graining

## Abstract

Microtubules are one of the most important components in the cytoskeleton and play a vital role in maintaining the shape and function of cells. Because single microtubules are some micrometers long, it is difficult to simulate such a large system using an all-atom model. In this work, we use the newly developed convolutional and K-means coarse-graining (CK-CG) method to establish an ultra-coarse-grained (UCG) model of a single microtubule, on the basis of the low electron microscopy density data of microtubules. We discuss the rationale of the micro-coarse-grained microtubule models of different resolutions and explore microtubule models up to 12-micron length. We use the devised microtubule model to quantify mechanical properties of microtubules of different lengths. Our model allows mesoscopic simulations of micrometer-level biomaterials and can be further used to study important biological processes related to microtubule function.

## Introduction

Microtubule (Fratzl and Weinkamer, 2007; Conde and Caceres, 2009) is one of the primary components of the cytoskeleton which is responsible for the morphology of eukaryotic cells. The building blocks of microtubules are α- and β-tubulins which assemble into a hollow cylinder structure with grooves. The length of a single microtubule can reach 10 µm and they are associated with each other in a hierarchical organization such as in the axons (Lazarus et al., 2015; Soheilypour et al., 2015). A microtubule is generally composed of 13 protofilaments (Kollman et al., 2010) lying side by side along the longitudinal direction, where the position of each protofilament shifts slightly to each other. The mechanical properties (Franz et al., 2020) of microtubules play important roles in sustaining the cell shape, executing the mitotic division and related physiological functions. For instance, they serve as tracks for the motor proteins kinesin (Gittes et al., 1996) to transport the substances required for cells.

In order to uncover the microscopic mechanisms of microtubules for transporting substances in cells, much effort has been put into measuring the biomechanical properties (Dye et al., 1993; Gittes et al., 1993; Venier et al., 1994; Kurachi et al., 1995; Felgner et al., 1996; Vinckier, 1996; Wagner et al., 1999; Kis et al., 2002; Le Goff et al., 2002; VanBuren et al., 2002; de Pablo et al., 2003; Janson and Dogterom, 2004; Pampaloni et al., 2006; Schaap et al., 2006; Hawkins et al., 2013; Cross, 2019; Szatkowski et al., 2019) of microtubules associated with their function, such as Young’s modulus *E*, the flexural rigidity *κ*, and the persistence length *l*_p_. Previous studies have exploited a variety of advanced techniques including thermal fluctuation (Gittes et al., 1993), hydrodynamic flow (Venier et al., 1994), atomic force microscopy (Vinckier, 1996), and optical tweezers (Felgner et al., 1996) to measure these mechanical quantities. So far, reported Young’s modulus *E*, the flexural rigidity *κ*, and persistence length *l*_p_ for microtubules with different lengths span a large range of 0.31–7.0 GPa (Kurachi et al., 1995; Vinckier, 1996; Kis et al., 2002; de Pablo et al., 2003; Schaap et al., 2006; Kis et al., 2008), 0.37–4.0 × 10^−23^ Nm^2^ (Gittes et al., 1993; Venier et al., 1994; Kurachi et al., 1995; Felgner et al., 1996), and 2.0–6.3 mm (Gittes et al., 1993; Venier et al., 1994; Janson and Dogterom, 2004; Pampaloni et al., 2006), respectively. Kis et al. (Kis et al., 2002) first reported that the observed phenomenon of the measured persistence length was length-dependent, caused by the anisotropic association of heterodimeric α- and β-tubulins. This finding was further demonstrated by Pampaloni et al. (Pampaloni et al., 2006) using the single-particle tracking method and they observed that the persistence length *l*_p_ increased for short lengths varying from 2.6 to 47.5 μm. It approached a constant value of *l*_p_^∞^ = 6.3 mm for lengths beyond 21 μm. All measured results unambiguously imply that microtubules do not follow the behavior of an ideal elastic rod and fail to be described with the worm-like chain model (Kroy and Frey, 1996; Taute et al., 2008) based on the isotropic hypothesis.

Theoretical modeling of microtubules (Kasas et al., 2004a; Kasas et al., 2004b; Molodtsov et al., 2005; VanBuren et al., 2005; Schaap et al., 2006; Deriu et al., 2007; Heuvel et al., 2007; Deriu et al., 2008; Dima and Joshi, 2008; Deriu et al., 2010; Sept and MacKintosh, 2010; Wells and Aksimentiev, 2010; Grafmuller and Voth, 2011; Ji and Feng, 2011a; b; Peter and Mofrad, 2012; Grafmuller et al., 2013; Theisen et al., 2013; Kononova et al., 2014; Lazarus et al., 2015; Soheilypour et al., 2015; Havelka et al., 2017; Stevens, 2017; Szatkowski et al., 2019; Tong and Voth, 2020) faces a huge challenge due to the micrometer length. All-atom molecular dynamics (MD) simulations have been primarily used to investigate the atomistic details of the interactions between the α- and β-tubulins (Deriu et al., 2007; Deriu et al., 2008; Sept and MacKintosh, 2010; Wells and Aksimentiev, 2010; Grafmuller and Voth, 2011; Grafmuller et al., 2013; Havelka et al., 2017) for short microtubules. Recent all-atom simulations (Tong and Voth, 2020) reached a length scale of a 1 μm microtubule including 3 protofilaments. Additionally, coarse-grained (CG) models (Dima and Joshi, 2008; Deriu et al., 2010; Ji and Feng, 2011a; Ji and Feng, 2011b; Theisen et al., 2013; Kononova et al., 2014; Lazarus et al., 2015; Soheilypour et al., 2015; Stevens, 2017; Szatkowski et al., 2019) have also been used to model longer microtubules. For instance, Deriu et al. (Deriu et al., 2010) constructed a 350 nm long microtubule to estimate its mechanical properties based on the elastic network model (Bahar et al., 1997; Atilgan et al., 2001; Xia and Lu, 2012; Xia et al., 2013a). Nevertheless, their model was still far shorter than the microtubules measured in experiments (Pampaloni et al., 2006). In some special CG models, each tubulin monomer of a microtubule was even represented by one CG bead (Ji and Feng, 2011a; Ji and Feng, 2011b; Lazarus et al., 2015) so that the whole microtubule model could reach the micrometer length. Alternatively, other theoretical methods based on continuous mechanics such as the finite element method (Kasas et al., 2004a; Kasas et al., 2004b; Schaap et al., 2006) were also used to evaluate the mechanical properties of macromaterials. However, these methods had an obvious disadvantage that they could not reflect the correct surface contour of microtubules, especially for the functional grooves serving as binding sites to interact with other proteins.

Ultra-coarse-grained (UCG) models (Dama et al., 2013; Davtyan et al., 2014; Dama et al., 2017; Zhang et al., 2017a; Jin et al., 2018; Jin and Voth, 2018) have been rapidly developed in recent years and applied to simulate the functions of biomolecular complexes such as the mechanical properties of F-actin (Katkar et al., 2018), the assembly of HIV capsids (Grime et al., 2016), and two-state conformational changes of proteins (Zhang et al., 2020). The bead resolution of UCG models is lower than the high-resolution models, with one UCG bead usually representing more than a few residues or even secondary structure elements. Previously, Voth and coworkers (Zhang et al., 2008; Zhang et al., 2009) developed the essential-dynamic coarse-graining (ED-CG) method to optimize the UCG representation (Sinitskiy et al., 2012) of proteins and later extended the framework to the density-based ED-CG method (Zhang and Voth, 2010). Xia and coworkers (Li et al., 2016a; Li et al., 2016b; Zhang et al., 2017b) developed a stepwise local iterative optimization-based fluctuation-maximization coarse-graining method to derive the optimal UCG representation (Wu et al., 2020) of large biomolecules. Further, Zhang et al. (Zhang et al., 2019) developed the convolutional and K-means coarse-graining (CK-CG) method to derive the UCG representation of biomolecules directly from the low-resolution cryo-EM density data (Ming et al., 2002; Esquivel-Rodriguez and Kihara, 2013), which allows the construction of models without any atomistic details needed further. Next, the UCG beads could be parameterized using the energy potential of the Multiscale Virtual Particle-based Anisotropic Network Model (MVP-ANM) (Xia et al., 2013b; Opron et al., 2014; Xia, 2017) for further functional studies. In the previous study (Zhang et al., 2019), we have validated the combined CK-CG and MVP-ANM methods in constructing UCG model for a correct estimation of the biomechanical properties of short segments F-actin and collagen fibrils. In this work, we attempt to verify the usefulness of UCG models in simulating the mechanical properties such as the length-dependent persistence length of long microtubules and expect to apply it to the simulation more complicated and hierarchically organized biomaterials (Zapp et al., 2020).

## Computational and Simulation Details

### Data Preprocessing and CK-CG Method

**FIGURE 1:**
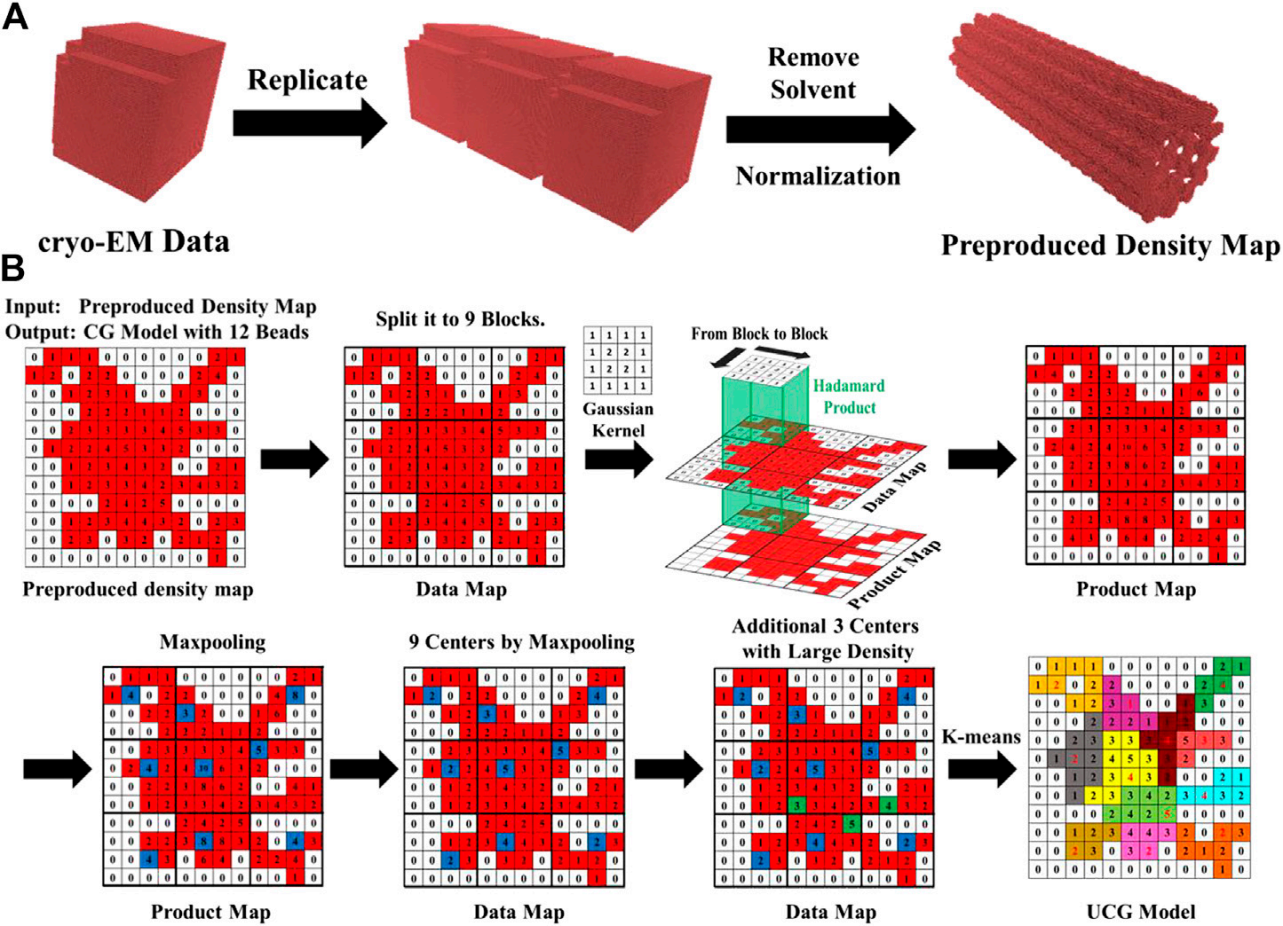
**(A)** The data reprocessing includes replicating the density, removing solvent, and normalizing density. **(B)** The process of coarse-graining cryo-EM data into 12 UCG beads using the CK-CG method. The density map is first divided into 9 blocks. The Hadamard product for each block is obtained by using a Gaussian kernel and the subsequent max-pooling generates the initial positions of 9 UCG beads. The positions of the remaining 3 UCG beads are determined by other maximal densities in the blocks. Then, the 12 positions serve as the centers for clustering to yield the final UCG beads.

The density file EMD-4043(Grange et al., 2017) of microtubule used for model construction was downloaded from the cryo-EM data bank online. The original length of microtubule in the file EMD-4043 is only 32 nm, in a resolution of 18.5 Å. In order to construct long microtubules, we replicated the density data shown in Figure 1A along the longitudinal direction and extended the 32 nm length to the different lengths ranging from 1 to 12 μm. Then, we removed the density of solvents from the raw data with a technique described in the previous work (Zhang et al., 2019) and evaluated the normalized density µ^s^ (r) according to µ^s^ (r) = (µ(r) − µ_min_)/(µ_max_ − µ_min_), where µ (r) denotes the cryo-EM density data. After the preprocessing on data, the remaining densities are positive and ready as the input for coarse-graining by using CK-CG method.

Coarse-graining density data is actually to cluster the adjacent discretized density data into a group of single UCG particles. In the conventional K-means method, the positions of center points in initial guess are generated randomly, which renders the convergence quite slow. In the CK-CG method, we utilized an idea similar to the Convolutional Neutral Network model (Li et al., 2019) to generate the initial guess for clustering, including the steps of the domain partition, density convolution, and max-pooling, displayed in Figure 1B. In more detail, the cryo-EM density data is firstly divided into a series of 3D block matrixes with the voxel *b*_*ijl*_, where the subscripts *i*, *j*, and *l* denote dimensions. In the next convolution operation, we used a 3D Gaussian kernel matrix with the voxel *k*_*ijl*_ to execute a Hadamard product based convolution operation and saved the matrix values *h*_*ijl*_.

In the subsequent max-pooling, the position corresponding to the largest density value in each block was chosen as the initial position of UCG bead for clustering. It has to be mentioned that if the expected number of UCG beads *N* is larger than the number of divided blocks *M*, the excess number of (*N*-*M*) beads might be automatically assigned into the blocks with largest values. After generating the initial guess, the clustering using K-means was carried out until the convergence was achieved. More details about the procedure of CK-CG workflow are described in the previous work (Zhang et al., 2019).

### Multiscale Virtual Particle-Based Anisotropic Network Model

UCG beads derived from CK-CG could be connected with harmonic springs and parameterized to be the MVP-ANM model developed by Xia et al. (Xia et al., 2013b; Opron et al., 2014; Xia, 2017). The energy function *E*_MVP−ANM_ is a sum of harmonic interactions as shown in Eq. 1, where *γ*_*ij*_ is a heterogeneous force constant, *d*_*ij*_ and $d_{ij}^{0}$ denote the instantaneous and equilibrium distances of pairwise UCG beads *i* and *j*, and $H\left(d_{c}-d_{ij}^{0}\right)$ is a Heaviside function depending on a cutoff distance $d_{c}$.

$$E_{\text{MVP}-\text{ANM}}=\sum_{i}\sum_{j>i}\gamma_{ij}{\left(d_{ij}-d_{ij}^{0}\right)}^{2}\cdot H\left(d_{c}-d_{ij}^{0}\right)$$ (1)

The parameter $\gamma_{ij}$ contains both the contributions from the density and distance of pairwise UCG beads *i* and *j*, with the expression shown in Eq. 2:

$$\gamma_{ij}=A\cdot \left(1+m_{i}\right)\left(1+m_{j}\right)\cdot e^{-d_{ij}^{2}/R^{2}}$$ (2)

**FIGURE 2:**
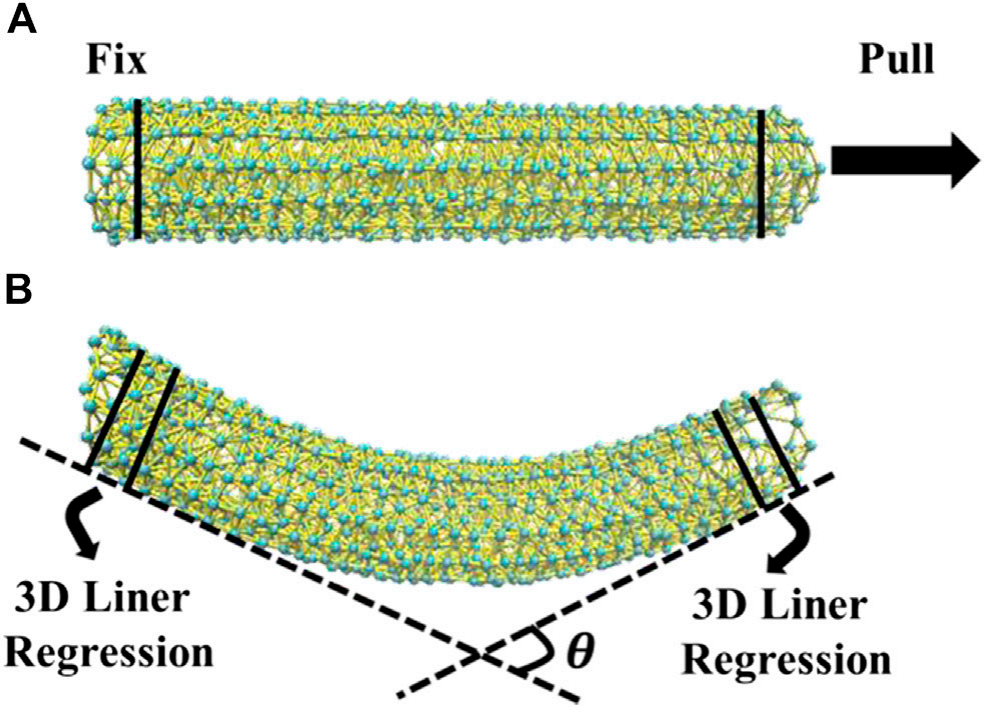
**(A)** An illustration of force-clamped MD simulation for measuring Young’s modulus of microtubule. **(B)** The estimation of persistence length using a method of 3D linear regression based on thermal fluctuation.

The parameter *A* is a scaling factor to control the mechanical properties of whole model and can be determined by fitting the experimental Young’s modulus using force-clamped MD simulation (Isralewitz et al., 2001; Gräter and Grubmuller, 2007) as shown in Figure 2A. The term of $\left(1+m_{i}\right)\left(1+m_{j}\right)$ is a function representing the mass contributions from the beads *i* and *j*. The distance contribution is defined as a Gaussian function in which *R* is a parameter to control the bond strength of pairwise beads *i* and *j*, and it is only taken into account if the equilibrium distance $d_{ij}^{0}$ is less than the chosen cutoff $d_{c}$. A minimal cutoff can be determined through MD simulations by judging whether the structures of microtubules remain stable all the time. Please see the details for determining a minimal $d_{c}$ described in *Parameterization of UCG Model* below. Additionally, the parameter $\gamma_{ij}$ decays significantly as $d_{ij}>3R$. Thus, we define the magnitude of *R* to be one-third of the minimal cutoff of *d*_*c*_.

### Evaluation of Persistence Length From UCG MD Simulation

We performed the UCG MD simulation of microtubules at 300 K and calculated the persistence length according the definition in Eq. 3:

$$\langle \cos\theta \rangle =e^{-L/lp}$$ (3)

where 〈cos⁡θ〉 means the average value of the cosine of *θ* (0≤θ≤π/2) and *θ* denotes the intersection angle of two tangent lines at the positions of the head and tail of microtubules. In order to calculate it, we recorded the Cartesian coordinates of all the beads every 12 ps in UCG MD simulation, and obtained a number of frames for further analysis. For the structure in each frame, we used a 3D liner regression method in Figure 2B to solve Eq. 3 and derived the persistence length from the calculated average value of 〈cos⁡θ〉. All the UCG MD simulations mentioned above were carried out with using the software LAMMPS (Plimpton, 1995) in the NVT ensembles at 300K. The Langevin dynamics simulation (Grønbech-Jensen, 2019) was performed under the nonperiodic condition with the damping factor being 10.0 ps. The time step of simulation is chosen to be 10.0 fs for the simulation of Young’s modulus. For the estimation of persistence length from a long time UCG simulation, since the lightest bead in the UCG model is around 6.0 kg/mol, nearly 6000 times greater than H atom, we chose a moderate value of 3.0 ps for the integration steps. The UCG MD simulation for a single microtubule with micrometer length could reach the time scale of 60 ms.

It has to be mentioned that the persistence length of microtubules was frequently estimated from the bending measurements in experiments (Kurachi et al., 1995; Felgner et al., 1996; Kis et al., 2008) and simulations (Kononova et al., 2014; Wu and Adnan, 2018). However, our UCG model is not suitable for being used in a direct simulation of bending deformation, since the MVP-ANM potential used in our model is harmonic and invalid in large deformation. A simulation of the complicated process such as the dissociation and association of tubulins from microtubule requires a more complex potential such as a Gō-like potential (Zhang et al., 2017a; Poma et al., 2017; Poma et al., 2018; Zhang et al., 2020).

## Results and Discussions

### Coarse-Graining Microtubules With Different Bead Resolutions and Lengths

Previously, we have constructed a UCG model for a short 160 nm microtubule (Zhang et al., 2019). In this work, we attempt to coarse-grain the microtubules with the lengths from 1 to 10 μm which are much longer than the previous one by 1 or 2 orders of magnitude. In order to demonstrate the efficiency of CK-CG over the conventional K-means methods, we test its time costs in coarse-graining the microtubules into different numbers of beads and lengths.

**FIGURE 3:**
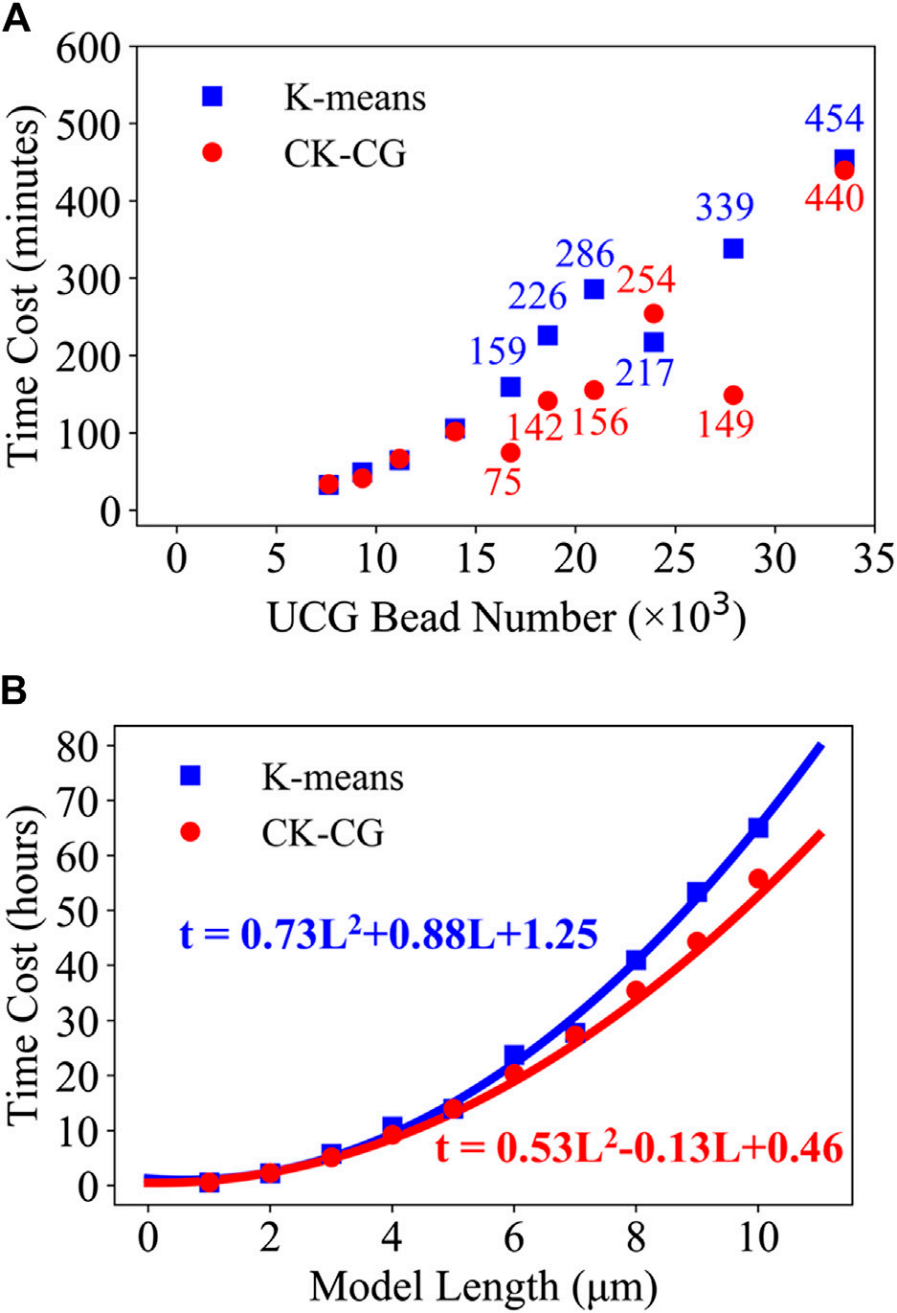
The time cost for building models with **(A)** different numbers of UCG beads and **(B)** different lengths. The blue squares and red dots represent the results from the K-means and CK-CG methods, respectively. The integers in plot **(A)** denote the exact time cost in minutes. In the plot (b), the set of data are fitted with two quadratic curves, respectively.

The first test given is the time cost for coarse-graining a 1 μm microtubule UCG model into different numbers of UCG beads. The test was done by running C++ language codes with one CPU on a CentOS platform. As shown in Figure 3A, the derived numbers of UCG beads for a fixed-length microtubule range from 7609 to 33480 beads per micrometer, corresponding to the averaged masses of UCG beads from 22.0 to 5.0 kg/mol. Figure 3A shows that the performances of the CK-CG and K-means methods are similar to each other in deriving the smaller numbers of UCG beads such as 7009, 9300, 11160, and 13950, whereas they exhibit distinct difference for large UCG numbers. The time cost of coarse-graining into 16740, 18600, 20925, and 27900 UCG beads by using CK-CG is almost half of that of K-means. For instance, coarse-graining into 27900 beads by CK-CG needs merely 149 minutes, while the K-means method costs 339 min. Also, it is seen that the time costs of both methods for deriving 23914 and 33480 UCG beads are comparable to each other. However, no case in which K-means was obviously superior to CK-CG was observed in our ten tests. Thus, the performance of CK-CG in coarse-graining a fixed-length microtubule into different beads outperforms the K-means method by 47.4 ± 44.4 hours with 95% confidence interval, from the statistical perspective. In this test, no simple correlation such as the linear relationship was found to exist between the time cost and the number of coarse-grained beads.

Next, we tested how the time cost changes with the increased lengths of microtubules under the same computational condition. The UCG models were built with the constraint of 7609 beads per micrometer, with the microtubule lengths changing from 1 to 10 μm. Figure 3B shows the comparison of time cost of using CK-CG and K-means methods for coarse-graining. It is surprisingly observed that the time cost exhibits a regular quadratic relationship with the increased length. It is found that the performances of two methods are nearly the same as the microtubules are short below 5 μm. Beyond that, the difference of time cost between them gradually becomes large with the increased length of microtubule. At the length of 10 μm, the CK-CG method saves 10 h more than K-means to optimize a total number of 76090 UCG beads. This comparison indicates that a good initial guess of center positions is important for accelerating the convergence of data clustering, especially for the biomolecules with the length at large scale.

### Determination of a Minimal Model for Microtubule

**FIGURE 4:**
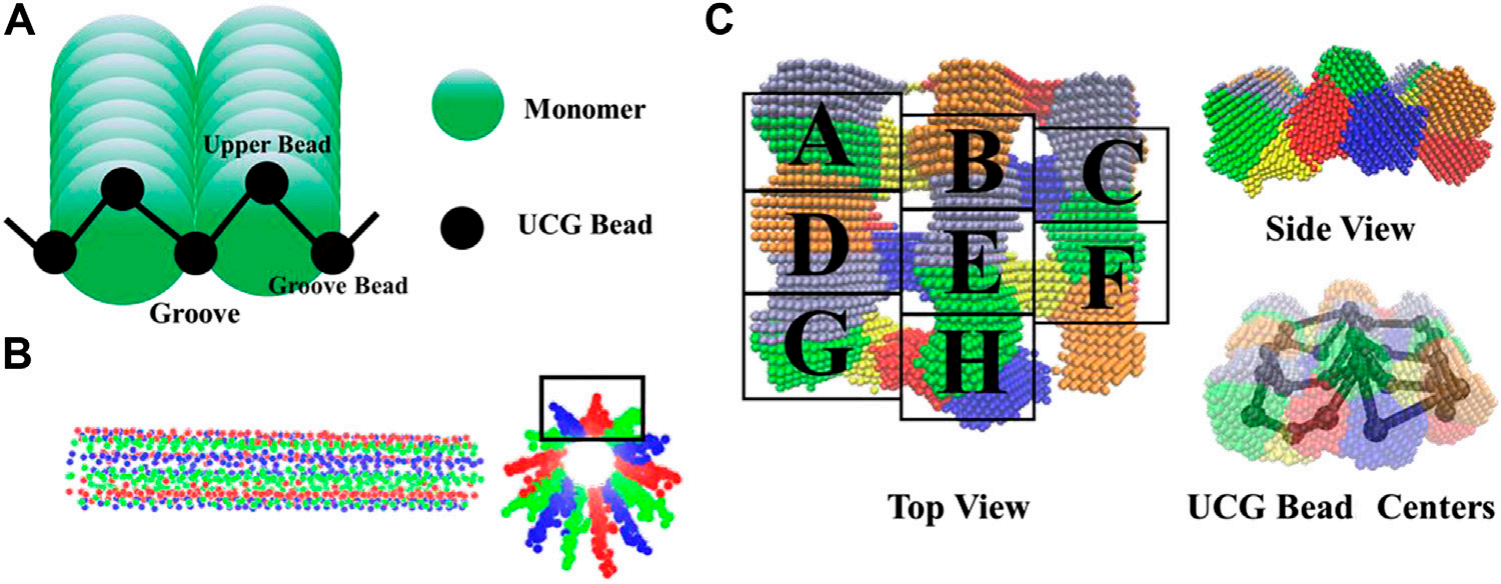
**(A)** An illustration of the minimal number of UCG beads required for maintaining the grooves of microtubule. **(B)** The transverse and longitude views of the built models. **(C)** Zoomed top and side views of the density map of 8 adjacent tubulins, labeled with the upper cases A-H. The different colors denote the density regions which are clustered into the UCG beads, with the corresponding UCG bead centers shown.

After validating the efficiency of CK-CG for coarse-graining micrometer microtubules, we are ready to construct a minimal model of microtubule for its mechanical simulation. The so-call “minimal model” must meet two requirements for functional study: one is that the number of UCG beads for a given length is so minimal that it can reduce the simulation time to the maximal extent. Secondly, it is expected that this model is capable of maintaining the geometric feature of microtubule, especially for the functional grooves. According to the two requirements, we designed a minimal model with the possible minimal number of beads, which is illustrated in Figure 4.

As shown in Figure 4A, the groove along the longitudinal direction of a microtubule is formed by two adjacent protofilaments. The simplest and minimal UCG model to characterize the feature of a groove is to represent each tubulin with two connected beads, with one shared by two neighboring tubulins. Since the average molar mass of tubulin is 55 kg/mol (Fratzl and Weinkamer, 2007), the estimated average molar mass of UCG beads is 22.5 kg/mol. To derive the positions of UCG beads, we set the average mass with the value of 22 kg/mol in the coarse-graining by CK-CG, which lead to a number of 7609 beads per 1 μm in *Coarse-Graining Microtubules With Different Bead Resolutions and Lengths*. Figure 4B shows the side and transverse views of the resulting microtubule model, where each UCG bead is virtualized with solid dots and the 13 protofilaments are in different colors.

Figure 4C shows the zoomed top and side views of a segment of density map of 8 tubulins, labeled with the upper cases A-H, where the density points belonging to the same UCG bead centers are in the same color. From the top and the side views, we can see clearly that the bottoms of most grooves are well represented by one UCG bead, such as the density regions connecting the adjacent “AB”, “DE”, or “BC” tubulins. Meanwhile, two adjacent tubulins such as “DG”, “BE”, or “EH” along a protofilament share the same beads. From the view of UCG beads, it can be judged that the UCG beads representing the groove bottoms of tubulins usually reside in a lower position, while the beads representing the tubulins along the protofilaments stay in a high position. Thus, the coarse-grained beads between two adjacent protofilaments maintain a geometric “V” shape along the contour surface of the microtubules, which is potentially important for functional studies including interacting proteins.

### Parameterization of Ultra-Coarse-Grained Model

**FIGURE 5:**
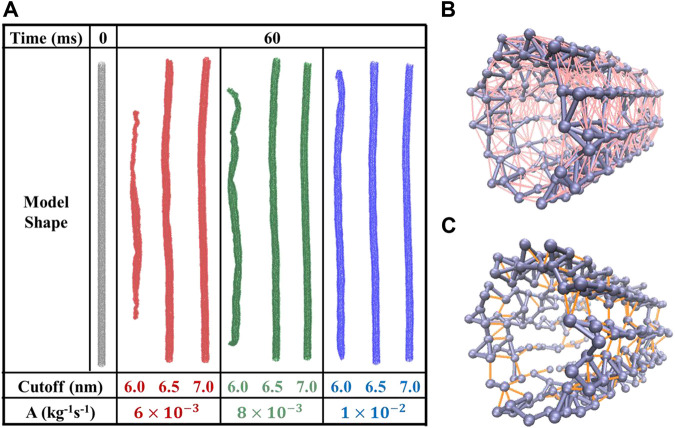
**(A)** The structures of constructed UCG models for microtubules at the initial simulation time and after 60 ms. The cutoff distances adopt the values of 6.0, 6.5, and 7.0 nm for three models, with the tested parameter *A* adopting 6 × 10^−3^, 8 × 10^−3^, and 1 × 10^−2^ kg^−1^ s^−1^, respectively. **(B)** The UCG bond distances below 4.4 nm are colored in gray, while those bonds in 4.4–6.6 nm are in pink. **(C)** The UCG bonds below 4.0 nm in gray and those in 4.0–4.4 nm are colored in orange.

Using the potential energy of MVP-ANM model of Eq. 1 to describe the interaction between pairwise UCG beads requires determining the two parameters *R* and *A* in Eq. 2. As mentioned in *Multiscale Virtual Particle-Based Anisotropic Network Model*, the parameter *R* could be defined as one-third of minimal cutoff distance *d*_*c*_. In a word, we need to determine a minimal cutoff at first. As shown in Figure 5A, we constructed the UCG models with the cutoff set as the tentative values of 6.0, 6.5, and 7.0 nm, respectively. Meanwhile, the parameter *A* adopts the empirical values of 6.0 × 10^−3^, 8.0 × 10^−3^, and 1.0 × 10^−2^ kg^−1^·s^−1^. After the 60 ms MD simulation, we found that all the three microtubule models with the cutoff of 6.0 nm have collapsed and their structures could not keep stable anymore. Besides, the UCG models with the cutoffs of 6.5 and 7.0 nm are stable in the long time MD simulation and maintain their tube shape well. The simulation results also indicate that the stabilities of microtubules did not depend on the choices of magnitudes of the parameter *A*.

To rationalize the choice of a cutoff of 6.5 nm, we analyze the number of harmonic bonds formed between pairwise UCG beads. In Eq. 2, the strength of harmonic bonds is weakened with an increasing distance *R*. When *d*_*c*_ adopts the cutoff 6.5 nm, we find that numerous bonds that describe strong interactions are defined between UCG beads, shown as gray lines in Figure 5B (Ji and Feng, 2011a; Kononova et al., 2014). Within the distance between 4.4 and 6.6 nm (2*R*–3*R*), bonds describing nonbonded interactions are found (shown in pink), and they are roughly 40 to 150 times weaker than the bonds within 4.4 nm. In contrast, Figure 5C shows the formed bonds within 4.0 nm in gray and the bonds in 4.0–6.0 nm in orange, as the cutoff adopts 6.0 nm. It is obvious that the two adjacent protofilaments in the UCG model do not feature sufficient bonding to keep the whole cylinder structure stable. Thus, the reason of collapse with the cutoff 6.0 nm lies in the lack of sufficient pairwise interactions to maintain the microtubule’s stability.

Eventually, we chose the cutoff to be 6.5 nm for all models and the resulting *R* value is 2.2 nm. The scaling factor *A* is determined by fitting the longitudinal Young’s modulus of a 100 nm short microtubule. There are two reasons that we need to fit Young’s modulus to derive the parameter *A*. One is that the longitudinal Young’s modulus is an independent property and has no influence on the measurement of the persistence length according to Eq. 3. The second is that the longitudinal Young’s modulus is independent of the length *L* of microtubule and only intrinsically determined by the strength of force constant *γ*_*ij*_. Thus, the parameter *A* in Eq. 2 is tightly correlated with the longitudinal Young’s modulus.

**FIGURE 6:**
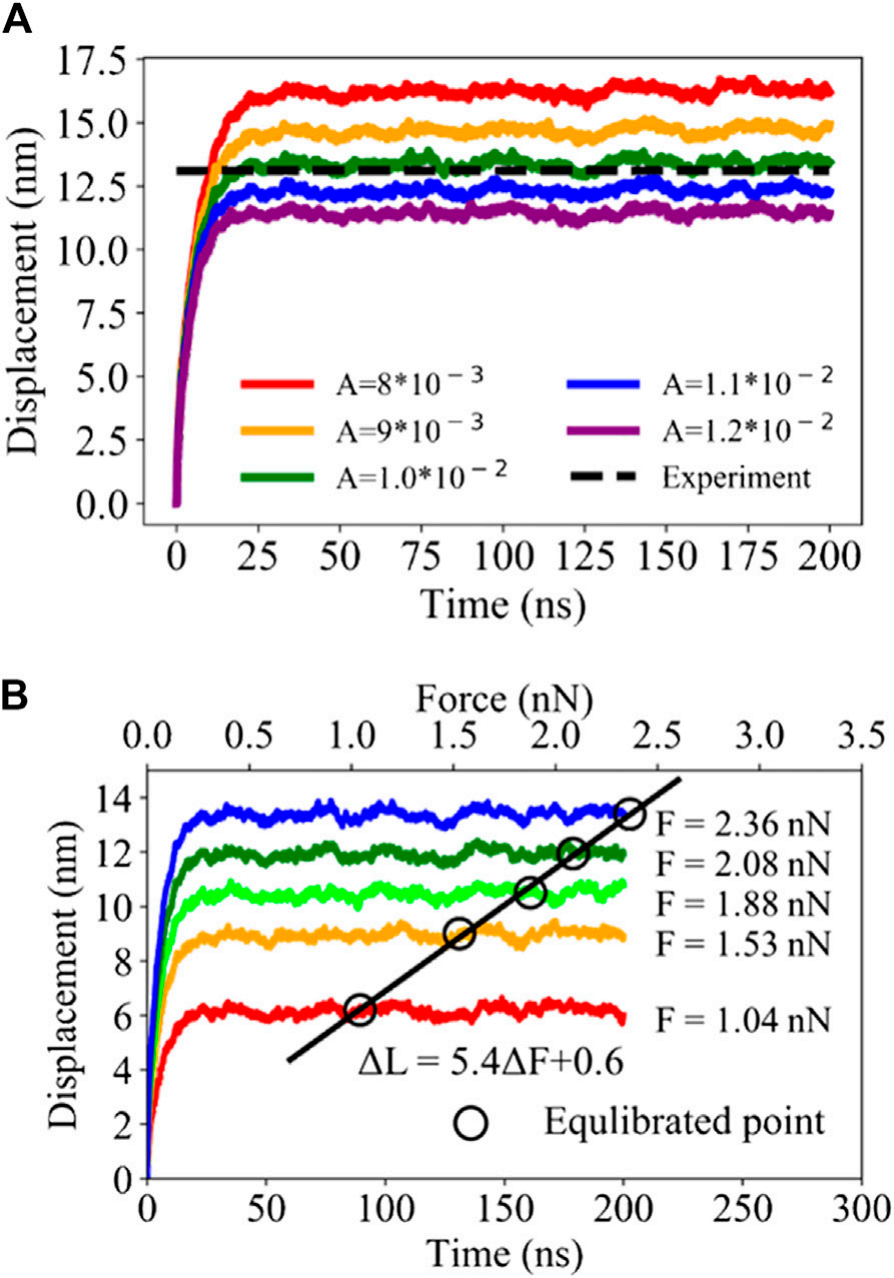
**(A)** The relationship of the terminal displacements of microtubule models with the different values used for the parameter *A* in the units of kg^−1^ s^−1^. The black dashed line represents the experimental displacement and the colorful curves denote the displacements of microtubule models changing with time under an external force of 2360 pN in force-clamp MD simulations. **(B)** The curves of the displacements of microtubule models under the different forces ranging from 1.04 to 2.36 nN, where the black hollow circles denote the average values of the end-to-end displacements sampled from 100–200 ns. The black line denotes the linear fitting of the circles.

Kis et al. (2002) reported that a microtubule had a longitude Young’s modulus of 100 MPa under 300 K. In our force-clamped MD simulation, one terminal of the 100 nm microtubule is fixed, while the other one is pulled with a constant force of 2.36 nN. In Figure 6A, different values of the parameter *A* are used for UCG models to measure the difference between the simulated displacements and the experimental result around 13.0 nm. As the parameter *A* adopts the value of 1.0 × 10^−2^ kg^−1^·s^−1^, the simulated displacement of microtubule reaches the experimental value well. So far, both the parameters *R* and *A* for the models of microtubules are determined and ready for the subsequent simulation of mechanical properties. In addition, we also performed force-clamped UCG MD simulations with the same parameters (A = 1.0 × 10^−2^ kg^−1^·s^−1^ and *R* = 2.2 nm) under different pulling forces ranging from 1.04 nN to 2.36 nN, as shown in Figure 6B. It can be observed that the equilibrated displacements have a good linear relationship with the applied forces. Based on the slope of the linear relationship, Young’s modulus is calculated to be 99.2 MPa, which reproduces Young’s modulus under different forces and verifies the validity of UCG model (Kis et al., 2002).

### Evaluation of Persistence Length

**FIGURE 7:**
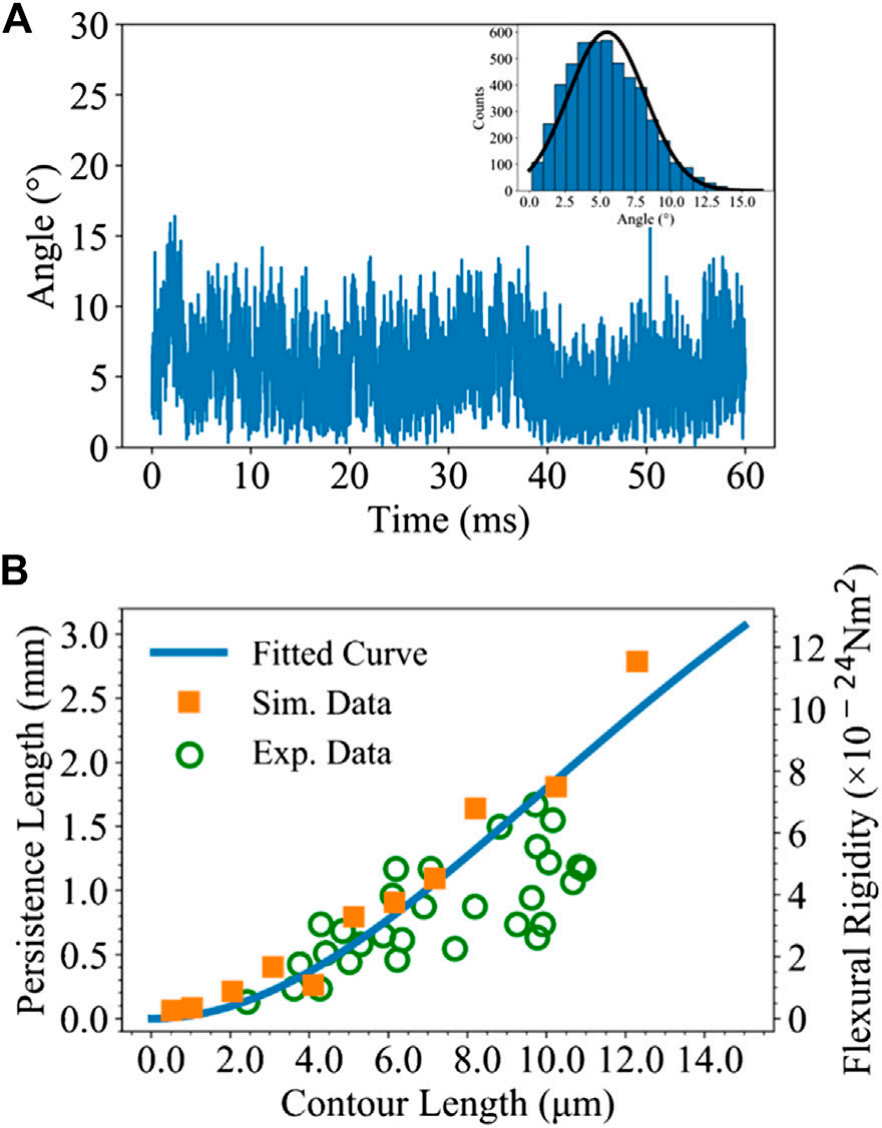
**(A)** The angle fluctuation of a 2 μm microtubule model within 60 ms. The inset displays the histogram distribution of angles with a black fitted Gaussian curve. **(B)** The orange solid squares show the relationship of the simulated persistence length and flexural rigidity with respect to the contour length, fitted with a blue curve based on Eq. 4. The green hollow circles denote the experimental measured results (Pampaloni et al., 2006). The simulation time ranges from microseconds to milliseconds for the different microtubules.

The persistence length of microtubule is evaluated by using MD simulation based on its thermal fluctuation. It should be noted that the experimental measurement of thermal fluctuation usually occurred at time scale of seconds. However, it is impossible to perform such a long time MD simulation in silicon, even by employing the UCG model. The previous simulation performed by Ding et al. (Ding and Xu, 2011) for evaluating persistence length from thermal fluctuation was 50 μs. In our UCG MD simulation, we used an integration step of 3 ps and the simulation time for microtubules with micrometer length could reach milliseconds. A number of MD trajectories were saved for further analysis. Figure 7A shows the recorded intersection angle of a 2 μm microtubule changing with time in MD simulation. It can be seen that the angle fluctuates between 0.0 and 17.0 degrees and the angles almost exhibit a Gaussian distribution in the inset. The data in Figure 7A suggest that our MD samplings are sufficient to evaluate persistence length.

Based on the sampled structures of microtubules with different lengths, we calculated the persistence lengths corresponding to different lengths according to Eq. 3. Figure 7B shows the change of calculated persistence length *l*_p_ changing with the contour length, with the longest microtubule being 12 µm. The calculated flexural rigidity *κ* according to the formula *κ* = *l*_p_ *k*_*B*_ *T* (Pampaloni et al., 2006) is plotted at the right side of y-axis, where *k*_*B*_ is the Boltzmann constant and *T* is the simulation temperature. The simulated data, denoted by the yellow squares, clearly verify that the persistence length increases nonlinearly with the length. During the simulated range of length from 0.5 to 12 µm, the variation tendency of *l*_p_ is in a good agreement with the experimental measurements (Pampaloni et al., 2006). In order to evaluate the magnitude of the persistence length *l*_p_^∞^, we fitted the data of calculated *l*_p_ with the microtubule length *L* according to Eq. 4 derived by Pampaloni et al. (Pampaloni et al., 2006). As shown in Eq. 4 below, the *l*_p_ has a complicated functional dependence on the *l*_p_^∞^ at infinity:

$$l_{p}=l_{p}^{\infty }{\left(1+\frac{3EI}{GkAL^{2}}\right)}^{-1}$$ (4)

where *E* is the longitude Young’s modulus and adopts the experimental value of 100 MPa (Kis et al., 2002) here, *A* is the area of cross section with 25 nm^2^ for a microtubule composed of 13 protofilaments, *I* is the area moment of the cross section with 16670 nm^4^, *k* is a geometric factor adopting 0.72, and *G* is the transverse sheer modulus. Figure 7B shows the fitted blue curve based on Eq. 4, similar to the fitted one in the previous work (Pampaloni et al., 2006). The persistence length *l*_p_^∞^ derived from our fitting is 7.0 mm, consistent with the experimental value of 6.3 ± 0.8 mm for long microtubules. They also calculated the term of (3*EI*/*GkA*)^0.5^ to be 21 μm (Pampaloni et al., 2006), which was considered as a threshold between short and long microtubules. We further compare our simulation results with the experimental data denoted by the hollow green circles below 12 μm (Pampaloni et al., 2006). It appears that our simulation results fit quiet well with the experimental measurements within the contour length of 10 μm. Besides, in the experimental measurement of microtubules up to 47.5 μm, the persistence length *l*_p_ seems to approach a constant beyond 21 μm. At present, our data obtained from UCG MD simulation based on the microtubule models with the lengths of 0.5–12 μm generated good results for *l*_p_ and *l*_p_^∞^ agreeable with known experimental results. In addition, the calculated values of the flexural rigidity *κ* for the length of 2–12 μm are in the range of 0.89–11.5 × 10^−24^ Nm^2^, falling in the range of the reported experimental values 3.7–40.0 × 10^−24^ Nm^2^ (Gittes et al., 1993; Venier et al., 1994; Kurachi et al., 1995; Felgner et al., 1996). However, a simulation of microtubules beyond 21 μm still faces a severe challenge memory requirement for calculating the interactions between pairwise particles in the UCG models.

## Conclusion

In this work, we introduce a systematic strategy to build a UCG model for microtubule with micrometer length from the cryo-EM density data. For the large microtubule with micrometer length, the CK-CG serves as an efficient coarse-graining method to derive its UCG representation. The comparison of time cost in coarse-graining the microtubules with the lengths from 1 to 10 μm demonstrates that CK-CG is superior to the conventional K-means in coarse-graining large systems. A reasonable minimal model was then determined for modeling the mechanical properties of microtubules. By means of using the MVP-ANM force fields, we parameterized the UCG beads and constructed the corresponding models for the study of mechanical properties.

We estimated the persistence length of microtubules with different lengths by using the force-clamp MD simulation based on thermal fluctuation. The obtained relationship between the persistence length and contour length accords well with the previous theoretical and experimental results, which demonstrates the validity of our method in modeling the mechanical properties of biomaterials. It is highlighted that our method has the advantage of being capable of constructing models directly from the low-resolution density data of cryo-EM, without any atomistic details required. Thus, it is particularly suitable for the functional study of macrobiomolecules for which only low-resolution cryo-EM data are available. Our model relies on low-resolution cryo-EM data, which is sufficient given our choice for an ultra-coarse-grained nature of our model. However, it remains to be examined if higher resolution cryo-EM structures can further improve models, even those on the very coarse scale such as the UCG model presented here. However, the current UCG model is still a sort of ENMs and it is limited to be valid in the study of biomechanical properties of biomaterials with small deformations under external forces. Future work will be invested into developing more physically meaningful energy potentials for UCG model and using our method to investigate longer microtubules and eventually the mechanical properties of a bundle of microtubules such as those present in axons.

## Data Availability

The raw data supporting the conclusions of this article will be made available by the authors, without undue reservation.
